# Wildlife inventory from camera-trapping surveys in the Azores (Pico and Terceira islands)

**DOI:** 10.3897/BDJ.8.e47865

**Published:** 2020-01-24

**Authors:** Lucas Lamelas-Lopez, Xose Pardavila, Isabel R. Amorim, Paulo A. V. Borges

**Affiliations:** 1 CE3C – Centre for Ecology, Evolution and Environmental Changes / Azorean Biodiversity Group and Universidade dos Açores, Angra do Heroísmo, Azores, Portugal CE3C – Centre for Ecology, Evolution and Environmental Changes / Azorean Biodiversity Group and Universidade dos Açores Angra do Heroísmo, Azores Portugal; 2 Área de Ecoloxía, Departamento de Bioloxía Celular e Ecoloxía, Universidade de Santiago de Compostela. Spain., Santiago de Compostela, Spain Área de Ecoloxía, Departamento de Bioloxía Celular e Ecoloxía, Universidade de Santiago de Compostela. Spain. Santiago de Compostela Spain

**Keywords:** Camera-traps, Vertebrates, Oceanic islands, Introduced species

## Abstract

**Background:**

The present publication provides a dataset from five camera-trapping sampling campaigns on two islands of the Azorean archipelago (Pico and Terceira islands), between 2013-2018. This dataset was obtained as a by-product of campaigns designed for different purposes. The sampling campaigns were designed to: (i) study the ecology of introduced mammals; (ii) assess the impact of introduced mammals on native birds (Azores woodpigeon - *Columba palumbus azorica* and Cory's shearwater - *Calonectris diomeda borealis*), through nest predation; and (iii) obtain information about the impact of vertebrates on agricultural systems, particularly on Azorean traditional vineyards. A total of 258 sites and 47 nests were sampled using camera traps. These sampling campaigns provided a large data series that allowed the creation of a vertebrate wildlife inventory.

**New information:**

We obtained a total of 102,095 camera-trap records, which allowed us to to identify 30 species of vertebrates: one amphibian, one reptile, 17 birds and ten mammal species. This represented 100% of the amphibians and terrestrial mammals, 58% of the breeding birds and 50% of the reptile species known for Pico and/or Terceira islands. Concerning the colonisation status of the species, we recorded 15 indigenous (native non-endemic or endemic) and three introduced bird species; all known terrestrial amphibians, reptiles and mammals in the Azores are introduced species. The data collected contribute to increasing knowledge on the distribution of vertebrate species on Pico and Terceira islands, where most existing records of some species were only available to Island level (e.g. mustelids and hedgehogs). None of the identified species was previously unknown to the study area.

## Introduction

Camera-trapping is commonly used to answer a variety of research questions in the fields of animal ecology, behavioural studies and conservation biology (O’Connell et al. 2011). It has also been used to assess the impacts of invasive species on native biodiversity (Hervías et al. 2012, Oppel et al. 2014) and the damage to wildlife in agriculture systems (Coates et al. 2010), as well as to address questions on spatial and temporal dynamics of animal populations (O’Connell et al. 2011). Although camera-traps are most often used to study medium- and large-sized terrestrial mammals (Tobler et al. 2008, Rovero et al. 2010), they have been successfully used to study arboreal mammals (Oliveira-Santos et al. 2008, Di Cerbo and Biancardi 2012) and small-sized species (MCCleery et al. 2014, Rendall et al. 2014, Taylor et al. 2014). Camera-traps are also useful for detecting rare or elusive species (Si et al. 2014).

Cameras are triggered by passing animals, allowing a record of animal presence and date and time of the detection (O’Connell et al. 2011). This allows the estimation of abundance and density (using capture-recapture models) for individually recognisable species (Karanth 1995, O’Connell et al. 2011). However, when it is impossible to distinguish specimens individually, camera-traps can also be used to calculate relative abundance indices of species (Rovero and Marshall 2009, O’Connell et al. 2011).

Camera-trapping has demonstrated to be one of the most efficient and low-cost sampling methods for faunal assessments, although it requires a large initial investment (Silveira et al. 2003, Lyra-Jorge et al. 2008, De Bondi et al. 2010). In particular, it is a very useful technique for wildlife inventories (Tobler et al. 2008, Rovero et al. 2010, Rovero et al. 2016) and monitoring (Yasuda 2004, Glen et al. 2013, Rendall et al. 2014), given its ability to generate large data series, recording the presence of target and non-target species.

In the Azores, very few studies have been done using camera-trapping to study vertebrates (but see, for example, Hervías et al. 2012 about nest predation). Here we aim to describe the main findings of the five sampling campaigns using camera-trapping to survey vertebrates, conducted between 2013 and 2018 in the Azores, namely on the islands of Pico and Terceira.

## General description

### Purpose

To provide a vertebrate inventory for the Azores (Pico and Terceira islands), based on data obtained as a by-product from five field sampling campaigns using camera-trapping.

### Additional information

Between 2013 and 2018, two sampling campaigns were conducted on Terceira island ("TER_13-15" survey) and on Terceira and Pico islands ("TER-PIC_18" survey) in order to study the ecology of introduced mammals. A third sampling campaign was performed between 2015 and 2017 in vineyards on Terceira island in order to evaluate grape consumption by vertebrate species ("Vineyards_15-17" survey). Additionally, between 2016 and 2018, two sampling campaigns were performed on Terceira island, in order to assess the impact of introduced mammals on native birds, on Cory's Shearwater (*Calonectris diomedea borealis*; "Calonectris_16" survey) and the Azores woodpigeon (*Columba palumba azorica*; "Columba_17-18" survey), through nest predation monitoring.

## Sampling methods

### Study extent

This dataset was obtained from different sampling campaigns performed between 2013 and 2018 in two islands of the central group of the Azores archipelago, Pico and Terceira Islands.

We described the study extent of the different sampling campaigns below:

- The survey "TER_13-15" was conducted between 2013 and 2015 on Terceira island, to investigate the ecology of introduced mammals. A total of 72 sites were sampled, but five sites were excluded due to camera failures. Each site was sampled during seven consecutive days.
- The survey "PIC-TER_18" was conducted in 2018 on Pico and Terceira islands, to investigate the ecology of introduced mammals. A total of 69 sites were sampled, with 33 and 34 sites, located in Pico and Terceira islands, respectively. Each site was sampled during ten consecutive days.
- The survey "Vineyards_15-17" was conducted in three consecutive years (2015, 2016 and 2017) in a vineyards area known as Protected Landscape Area of Biscoitos Vineyards, located in the North of Terceira island, to evaluate grape consumption by vertebrates. A total of 117 sites were sampled, with 20, 49 and 48 sites sampled during 2015, 2016 and 2017, respectively. Each site was sampled during seven consecutive days.
- The survey "Calonectris_16" was conducted in 2016 on Terceira island, to assess the impact of introduced mammals. A total of 17 nests of *Calonectris diomedea borealis* were sampled. Each nest was sampled during ten consecutive days.
- The survey "Columba_17-18" was conducted in 2017 and 2018 on Terceira island, to assess the impact of introduced mammals. A total of 30 nests of *Columba palumbus azorica* were sampled, with 9 and 21 sites sampled in 2017 and 2018, respectively. Each nest was sampled during ten consecutive days.

### Sampling description

All sites were sampled using camera-traps that were fixed to a tree or wooden stick. The sampling effort was measured as camera-trap days, i.e. the number of camera traps multiplied by the number of days that they remained active (Rovero et al. 2010). The sensitivity of the infrared sensor was configured to high to increase the species detection (O’Connell et al. 2011). Cameras were configured to take events with 30 seconds of delay between them, recording the date and time of each event. Cameras remained active 24 hours per day.

For the surveys "TER_13-15" and "PIC-TER_18" sampling sites were randomly selected, separated at least by 1 km. In each sampling site, one camera trap and a bait were deployed, 150-200 cm apart. Bait, consisting of meat or fish, fruit or vegetables and molasses, was used to increase the species detection (du Preez et al. 2014).

**Figure 1.**
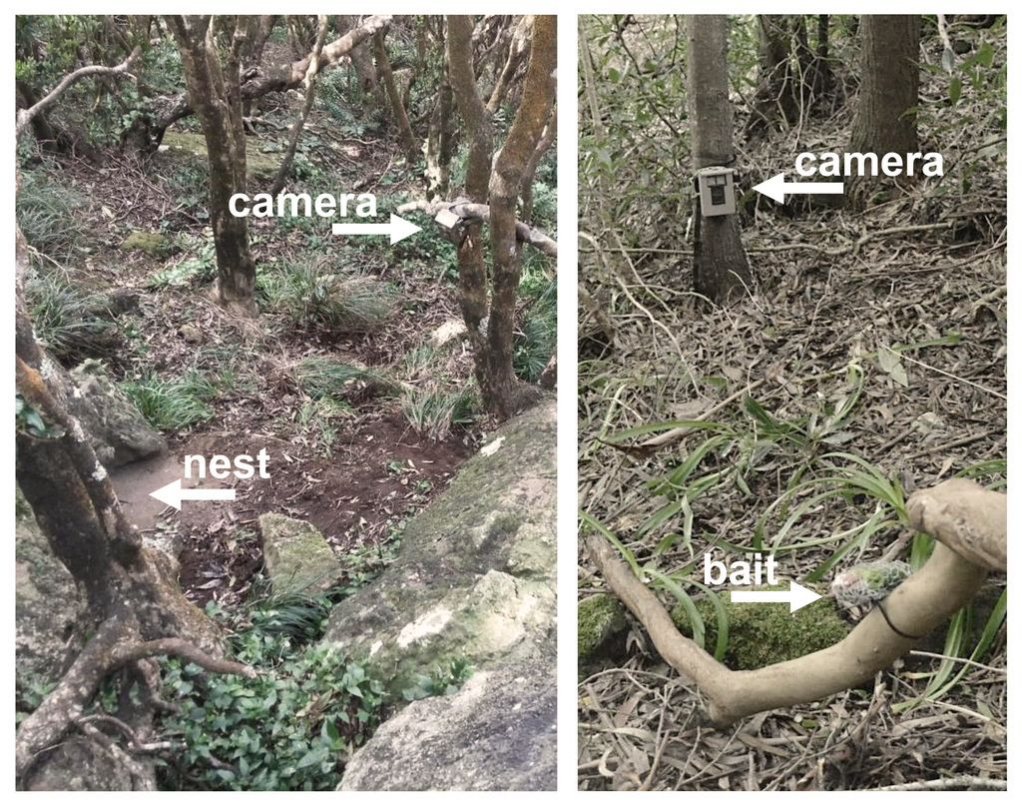
Examples of camera trap sampling sites. Left: camera facing a *Calonectris diomedea* nest; Right: camera trap facing a bait station.

For the surveys "Vineyards_15-17", "Calonectris_16" and "Columba_17-18", no bait was used. In the case of the "Vineyards_15-17" survey, sampling sites were selected at random, deploying one camera at each site, facing bunches of grapes. For "Calonectris_16" and "Columba_17-18" surveys, one camera was installed at 50-150 cm from the study nest (Fig. 1).

### Quality control

Taxonomic nomenclature followed the GBIF species profile and, for Azorean subspecies, we used the vertebrate checklist in Borges et al. 2010.

## Geographic coverage

### Description

Pico and Terceira islands, the Azores, Macaronesia, Portugal

### Coordinates

38.434491 and 38.7617777778 Latitude; -28.4228543692 and -27.1971972222 Longitude.

## Taxonomic coverage

### Taxa included

**Table:** 

Rank	Scientific Name	Common Name
class	Mammalia	Mammals
class	Aves	Birds
class	Reptilia	Reptiles
class	Amphibia	Amphibians

## Temporal coverage

**Data range:** 2013-9-08 – 2018-8-10.

## Usage rights

### Use license

Open Data Commons Attribution License

## Data resources

### Data package title

Wildlife inventory in the Azores using camera trapping

### Resource link

http://ipt.gbif.pt/ipt/resource?r=camera_trapping_azores

### Alternative identifiers

https://www.gbif.org/dataset/7d6b90d2-14c0-4ba6-9e45-449b56bab878

### Number of data sets

1

### Data set 1.

#### Data set name

Wildlife inventory in the Azores using camera trapping

#### Data format

Darwin Core Archive

#### Number of columns

39

#### Data format version

version 1

#### Description

The dataset was published in GBIF (see Lamelas-López et al. 2019). The following data table includes all the records for which a taxonomic identification of the species was possible. The dataset submitted to GBIF is structured as a sample event dataset, with two tables: event (as core) and occurrences. The data in this sampling event resource have been published as a Darwin Core Archive (DwCA), which is a standardised format for sharing biodiversity data as a set of one or more data tables. The core data table contains 2,308 records (eventID). One extension data table also exists with 108,186 occurrences. This large number of occurrences is a consequence of the fact that cameras were configured to take occurrences with 30 seconds of delay between them, recording the date and time of each record. Cameras remained active 24 hours per day. An extension record supplies extra information about a core record. The number of records in each extension data table is illustrated in the IPT link. This IPT archives the data and thus serves as the data repository. The data and resource metadata are available for downloading in the downloads section.

**Data set 1.** 

Column label	Column description
Table of Sampling Events	Table with sampling events data
eventID	Identifier of the events, unique for the dataset
samplingProtocol	The sampling method used
sampleSizeValue	The number of days that the cameras remain active in each sampling
sampleSizeUnit	The unit of the sample size value
samplingEffort	The number of camera-trap days expended during an event
eventDate	Date when the event occurred
habitat	The habitat type in which the event occurred
locationID	An identifier of the camera location
island	Name of the island on which camera location occurs
country	Country in which camera location occurs
countryCode	ISO code of the country in which camera location occurs
stateProvince	Name of the region in which camera location occurs
decimalLatitude	The geographic latitude, in decimal degrees
decimalLongitude	The geographic longitude, in decimal degrees
geodeticDatum	The reference point for the various coordinate systems used in mapping the earth
coordinateUncertaintyInMetres	Uncertainty of the coordinates, in metres
fieldNotes	Notes about the use or non-use of bait in the sampling sites
eventRemarks	Additional information supporting the survey code
Table of Occurrences	Table with species occurrences
id	Unique identifier
eventID	A unique identifier of an occurrence
ocurrenceID	Identifier of the event, coded as a global unique identifier
basisOfRecord	The nature of the data record
eventDate	Date when the event occurred
eventTime	Time of the day when the event occurred
organismQuantity	A number for the quantity of organisms
organismQuantityType	The unit used to quantify the organisms
occurrenceStatus	Information about the presence/absence of a taxon at a camera location
kingdom	Kingdom name in which the taxon is classified
phylum	Phylum name in which the taxon is classified
class	Class name in which the taxon is classified
order	Order name in which the taxon is classified
family	Family name in which the taxon is classified
genus	Genus name in which the taxon is classified
specificEpithet	Species name in which the taxon is classified
infraspecificEpithet	Subspecies name in which the taxon is classified
scientificName	The full scientific name including author and year
taxonRank	Lowest taxonomic rank of the event

## Additional information

**Results and Discussion**

**Figure 2.**
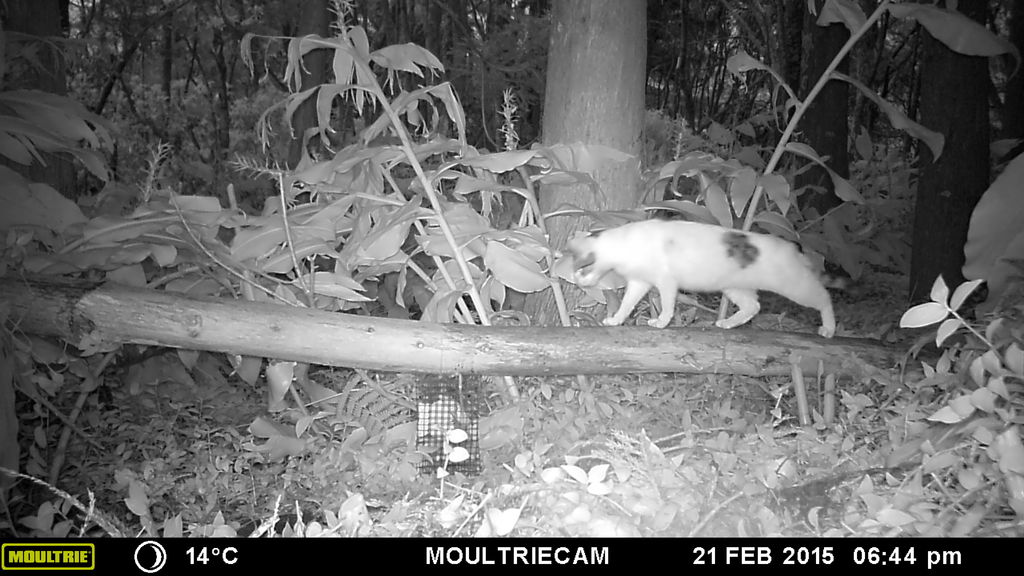
Example of a camera trap record. The record shows the presence of a cat (*Felis catus*) on 21 February 2015, at 06:44 pm.

A total of 102,095 records were obtained (see example in Fig. 2): two were amphibians, 12,072 reptiles, 61,329 birds and 28,692 mammals. The majority of records were obtained on Terceira Island (94,731) since most of the sampling campaigns occurred on this island. Additionally, for 11,203 records, species identification was not possible, although most individuals could unequivocally be assigned to the mammal order Rodentia (11,055). This dataset provides reliable records that contribute to increasing knowledge on the distribution of vertebrate species on Pico and Terceira islands, where most existing records of some species were only available to Island level (e.g. *Mustela furo*, *Mustela nivalis* or *Erinaceus europaeus*), according to current GBIF occurrence data. Spatial (e.g. habitat type) and temporal (date and hour of the record) information of the species are also included in the dataset resources.

A total of 30 species were identified: one amphibian, one reptile, 17 birds and ten mammal species (Table 1). According to the most recent available Azorean biota checklist (Borges et al. 2010), we recorded 100% of the amphibians and terrestrial mammals, 58% of the breeding birds and 50% of the reptile species known for Pico and/or Terceira islands. Cattle (e.g. cows) and invertebrate species (e.g. flies, bees or snails) were also recorded, but were excluded from the analysis and results.

The species with the highest number of records were the Azores Woodpigeon - *Columba palumbus azorica* (53,752), the black rat - *Rattus rattus* (40,756) and the Madeira lizard - *Teira dugesii* (24,074). In the case of the Azores Woodpigeon, the high number of records was due to the fact that adults remain in the nests for long periods, causing many camera captures.

Green frog - *Pelophylax perezi*, the European quail - *Coturnix coturnix*, Azorean buzzard- *Buteo buteo rothschildi*, Atlantic yellow-legged gull - *Larus michaellis atlantis*, common snipe - *Gallinago gallinago*, long-eared owl - *Asio otus* and grey wagtail - *Motacilla cinerea patriciae* were the least captured species (< 10 records).

In total, we recorded 15 indigenous (native non-endemic or endemic) and three introduced bird species (Table 1). All known amphibians, reptiles and terrestrial mammals found in Azores are introduced species (Borges et al. 2010). None of the identified species was previously unknown to the study area.

## Figures and Tables

**Table 1.** Number of events (photos or videos) and colonisation status of species recorded in different projects in Terceira and Pico, since 2013 until 2018, based on camera-trapping. Abbreviations: endemic subspecies of Azores (azo); endemic of Macaronesia (mac); introduced (int); native non-endemic (nat).

Class	Species	Status	TER_13-15	PIC-TER_18	Vineyards_15-17	Calonectris_16	Columba_17-18
Amphibia	*Pelophylax perezi* (López-Seoane, 1885)	int	0	2	0	0	0
Reptilia	*Teira dugesii* (Milne-Edwards, 1829)	int	56	985	11017	14	0
Aves	*Coturnix coturnix conturbans* Hartert, 1917	nat	0	0	1	0	0
Aves	*Calonectris borealis* (Cory, 1891)	nat	0	3	0	2015	0
Aves	*Buteo buteo rothschildi* (Swann, 1919)	azo	1	0	0	0	0
Aves	*Larus michahellis atlantis* Dwight, 1922	azo	4	0	0	0	0
Aves	*Gallinago gallinago* (Linnaeus, 1758)	nat	0	2	0	0	0
Aves	*Scolopax rusticola* Linnaeus, 1758	nat	16	102	0	0	0
Aves	*Columba livia domestica* Gmelin, 1789	int	1	0	23	100	0
Aves	*Columba palumbus azorica* Hartert, 1905	azo	47	6	40	0	53752
Aves	*Asio otus otus* (Linnaeus, 1758)	nat	7	0	0	0	0
Aves	*Chloris chloris aurantiiventris* (Cabanis, 1851)	int	9	0	2	0	0
Aves	*Fringilla coelebs moreletti* Pucheran, 1859	azo	60	117	88	1	8
Aves	*Serinus canaria* (Linnaeus, 1758)	mac	2	6	259	2	0
Aves	*Motacilla cinerea patriciae* Vaurie, 1957	azo	6	0	0	0	0
Aves	*Passer domesticus domesticus* Linnaeus, 1758	int	2	3	1544	0	0
Aves	*Sturnus vulgaris granti* Hartert, 1903	azo	10	0	0	1	0
Aves	*Sylvia atricapilla gularis* Alexander, 1898	azo	4	248	65	2	2
Aves	*Erithacus rubecula rubecula* (Linnaeus, 1758)	nat	25	92	6	6	0
Aves	*Turdus merula azorensis* Hartert, 1905	azo	300	1403	912	23	1
Mammalia	*Mustela nivalis* (Linnaeus, 1758)	int	14	1	13	2	0
Mammalia	*Mustela furo* (Linnaeus, 1758)	int	4	43	0	1	0
Mammalia	*Felis silvestris catus* Schreber, 1775	int	996	1042	41	10	30
Mammalia	*Canis lupus familiaris* Linnaeus, 1758	int	150	64	1	0	0
Mammalia	*Dama dama* (Linnaeus, 1758)	int	0	4	0	2	0
Mammalia	*Mus musculus* Linnaeus, 1758	int	83	3037	63	2	0
Mammalia	*Rattus norvergicus* (Berkenhout, 1769)	int	0	2134	0	0	0
Mammalia	*Rattus rattus* (Linnaeus, 1758)	int	0	20239	0	0	278
Mammalia	*Oryctolagus cuniculus* (Linnaeus, 1758)	int	369	3	4	0	0
Mammalia	*Erinaceus europaeus europaeus* (Linnaeus, 1758)	int	40	20	2	0	0
